# Counselling Needs of Sickle-Cell Anaemia Adolescents in Ekiti State, Nigeria

**DOI:** 10.4314/ejhs.v30i6.19

**Published:** 2020-11

**Authors:** Lateef Omotosho Adegboyega

**Affiliations:** 1 Department of Counsellor Education, Faculty of Education, University of Ilorin, Ilorin, Nigeria

**Keywords:** Counselling Needs, Sickle-cell Anaemia, Adolescents

## Abstract

**Background:**

Sickle-cell disease is an autosomal recessive genetic disorder of hemoglobin (Hb) structure and the most common of the hemoglobinopathies. Hence, this study investigated counseling needs of sickle-cell anaemia adolescents in Ekiti State.

**Methods:**

Descriptive survey design was adopted for this study. Purposive sampling technique was adopted to draw a total of 121 respondents. A questionnaire was used to collect data for the study. Mean and rank order were used to answer the research question while chi-square and Analysis of Variance (ANOVA) were used to test the hypotheses at 0.05 level of significance.

**Results:**

The findings revealed that counselling needs of adolescents with sickle-cell anaemia include counsellors are expected to encourage adolescents with sickle-cell anaemia to have confidence in self among others. The findings also revealed that there was a significant difference in the counselling needs of adolescents with sickle-cell anaemia based on gender while there was no significant difference in the counselling needs of adolescents with sickle-cell anaemia based on religion.

**Conclusion:**

The counselling needs of adolescents with sickle-cell anaemia include adolescents with sickle-cell anaemia easily comprehend the counselling therapy of counsellor among others. It was recommended that Government should offer standard health care for all adolescents with Sickle-cell disease.

## Introduction

Sickle-cell Disease (SCD) is an autosomal recessive genetic disorder of hemoglobin (Hb) structure and the most common of the hemoglobinopathies. While it usually results in anaemia, the primary symptomatic manifestation of SCD is pain. The most severe form of SCD, homozygous sickle-cell anemia (Hb SS) occurs when Hb S is inherited from both parents. Other genetic variants producing SCD include two forms of sickle-cell-beta thalassemia (Sβ° and Sβ+) and Sickle-cell-hemoglobin C (SC). Individuals with sickle-cell trait, i.e, heterozygotes for HbS, do not experience any adverse clinical consequences (except under acute hypoxic conditions, eg, exposure to high altitude without time to accommodate) and having had a selective advantage against malaria. As with most chronic diseases, depression and other psychiatric disorders are common in SCD. Alao and Cooley (1) noted that the rates of depression are similar to those found in other serious chronic medical disorders. It ranges from 18% to 44%, and are increased over rates in the general population even when one controls for illness-related physical symptoms.

Adolescents with sickle-cell disease may display physical manifestations of their illness. As a result of short stature, low muscle mass or jaundiced eyes and nail-beds, ridicule by peers and others are possible. This is particularly common in children 8 to 12 years of age (2,3). Dobbie and Meller (4) noted that some children with sickle-cell disease are frequently absent from school. These absences may be the result of a painful episode, hospitalization, outpatient visits and procedures or other illnesses. Frequent absences from school may result in incomplete class work and incomplete development of social skills. Students can feel disenfranchised from classroom activities and classmates. However, in this study, the age group of sickle-cell anaemia adolescents in Ekiti State, Nigeria are boys and girls between ages 13 and 19.

Counselling is an essential tool for effective interpersonal relationship for self-understanding as well as equitable adjustment to our environment. In support of this, Odebunmi (5) viewed it as encompassing the full range of personalized assistance given to the individual seeking to expand his self-understanding and his understanding of others. The guidance and counselling profession is one that is unique and of immense importance to mankind (6). Although it is practiced in a local form in many quarters, yet many people have not come to grasp with what it entails. It is necessary for adolescents with sickle-cell anaemia to be properly counseled in order to cater for any form of problems facing them. Hence, this study investigated the counselling needs of sickle-cell anaemia adolescents in Ekiti State, Nigeria.

## Methods

The research design that was employed for this study is descriptive survey. The descriptive nature of this design makes it suitable for this study since it is aimed at investigating the counselling needs of sickle-cell anaemia of adolescents in Ekiti State; the sickle-cell anaemia adolescents are between ages 13 and 19. The population for the study comprised all adolescents with sickle-cell disease in the sixteen Local Government Areas of Ekiti State. This included adolescents of various religious backgrounds. The adolescents include both males and females. According to the statistics provided by Ekiti State Ministry of Health (7), the total number of adolescents with sickle-cell disease in Ekiti State is approximately 2,309.

Purposive sampling was adopted to select adolescents with SCD in selected General Hospitals in Ekiti State University Teaching Hospital (ESUTH) and Federal Teaching Hospital (FTH), Ido-Ekiti. Out of approximately 2,309 SCD patients attending the selected hospitals in Ekiti State, an estimated sample size of 110 was made with an attrition rate of 10% (11) added to it, giving a total of 121 for the sample, in an agreement with Fishers formula for a population of less than 10,000.

A researcher-designed instrument was used to collect data from the respondents. The instrument is entitled “Counselling Needs of Adolescents with Sickle-cell Anaemia Questionnaire (CNASAQ)”. The questionnaire has two sections, A and B. Section A of CNASAQ elicited the respondents' personal data while section B contains 20 items that sought to measure the counselling needs of adolescents with sickle-cell anaemia. In order to ascertain the validity of the questionnaire, the draft of the questionnaire was given to five experts in counselling and social work Department, University of Ilorin, for vetting and advice. Sequel to their suggestions, necessary amendments were made. The reliability of the instrument was ascertained through test re-test; a co-efficient of 0.89 was obtained.

The objective of the study was clearly explained to the respondents by attaching informed consent forms to the instrument. Respondents' consents were sought to include them in the study and they were informed that participation in the study was voluntary and that they can opt out at any point in time. Respondents were assured of utmost anonymity and confidentiality of any information provided on the questionnaire form.

The data collected were analyzed using mean and rank order for the research question. Hypothesis 1 was tested using chi-square (χ^2^) while hypothesis 2 was tested using Analysis of Variance (ANOVA) at 0.05 level of significance.

This research question was raised based on the problem of the study: What are the counselling needs of adolescent with sickle-cell anaemia in Ekiti State, Nigeria?

The following null hypotheses were postulated to guide the conduct of the study:

1. There is no significant difference in the counselling needs of adolescents with sickle-cell anaemia based on gender.
2. There is no significant difference in the counselling needs of adolescents with sickle-cell anaemia based on religious affiliation.

## Results

The distribution of respondents by gender shows that 71(58.7%) of the respondents were males while 50(41.3%) of the respondents were females. This indicates that more males than females participated in the study. The distribution of respondents by religion shows that 2(1.7%) of the respondents were African Traditional Religion adherents, 107(88.4%) of the respondents were Christian and 12(9.9%) of the respondents were Muslims. This shows that respondents who were Christians participated more in the study. The distribution of respondents by age shows that 88(72.7%) of the respondents were 13–15 years in age, 27(27. 3%) of the respondents were 16–17 years and 6(5.0%) respondents were 18–19 years in age. This shows that respondents who were 13–15 years in age participated more in this study.

**Research Question 1**: *What are the counselling needs of adolescents with sickle-cell anaemia in Ekiti State, Nigeria?*

The mean and rank order (Table 1) shows the respondents' expression on the counselling needs of adolescents with sickle-cell anaemia. The table indicates that most of the items have the mean scores that are above the mid-cut off point of 2.5. This indicates that the respondents attested to the counselling needs of adolescent with sickle-cell anaemia listed above.

**Table 1:** Mean and Rank Order on the Respondents' Expression on Counseling Needs of Adolescents with Sickle-cell Anaemia

Item No.	Counselling Needs of Adolescents with Sickle-Cell Anaemia	Mean	Rank Order
1	The counsellor is expected to encourage me to have confidence in myself.	3.66	1^st^
13	I easily comprehend the counselling therapy of my counsellor.	3.64	2^nd^
6	I am always willing to disclose myself to the counsellor.	3.44	3^rd^
7	I always enjoy special consideration from my counsellor.	3.31	4^th^
2	The counsellor expects to guide me on choice of course.	3.17	5^th^
14	Counsellor encourages me to tolerate others.	3.12	6^th^
8	Counsellor counsel that been a sickle-cell do not call for shorten of life span.	3.07	7^th^
17	The counsellor connects me with another helping profession like psychologist, social workers.	3.05	8^th^
9	Counsellor educates the sickle-cell anemia that I should believe in God than in man.	2.88	9^th^
19	With all the counselling experience I had, am filled with joy.	2.75	10^th^
15	Counsellor tells about the reality of life challenges to face.	2.70	11^th^
18	The counsellor relates me with story that can make me feel like a complete human being.	2.67	12^th^
3	The counsellor expects to provide necessary guides for self fulfilment.	2.67	12^th^
11	Counsellor organize enlightenment program for the parent of adolescence living with sickle-cell anemia, to support their well being.	2.55	14^th^
10	Once the opposite sex genotype is not SS they can go ahead with their relationship.	2.43	15^th^
20	Counselling reduces negative thinking about my life.	2.42	16^th^
4	The counsellor expects to connect me with other necessary community resources.	2.40	17^th^
12	Counsellor counsel the adolescence with sickle-cell anemia on the purpose of this life.	2.07	18^th^
16	The counsellor always keeps me abreast of every new drug to be taken as a sickle-cell anemia.	2.05	19^th^
5	It is expected of a counsellor to always ready to give me attention.	1.87	20^th^

**Hypothesis One**: *There is no significant difference in the counselling needs of adolescents with sickle-cell anaemia based on gender.*

The testing of hypothesis one (Table 2) shows that the calculated χ^2^-value is 3.68 with a corresponding p-value of 0.03. This indicated that a significant difference exists in the respondents' counselling needs based on gender. Thus, the hypothesis was rejected. Since the calculated p-value is less than the critical p-value, the hypothesis which states that there is no significant difference in the counselling needs of adolescents with sickle-cell anaemia based on gender is rejected. The table also presents frequency of respondents based on gender and their mean score or response.

**Table 2:** Chi-square (χ^2^) Analysis Showing Difference in Respondents' Expression on Counselling Needs of Adolescents with Sickle-cell Anaemia Based on Gender

Gender	Counselling Needs			Total	Cal. p-value	Decision
Male	Observed	68	3	71	0.03	**H_01_** **Rejected**
	Expected	59.2	11.8	71.0		

Female	Observed	33	17	50		
	Expected	44.5	5.5	50.0		

Total	Observed	101	20	121		
	Expected	103.7	17.3	121.0		

* Sig. at p < 0.05

**Hypothesis Two**: *There is no significant difference in the counselling needs of adolescents with sickle-cell anaemia based on religion.*

The testing of hypothesis two (Table 3) shows that the calculated F-ratio of 0.84 is less than the critical F-ratio of 3.00 with a corresponding p-value of .432 which is greater than 0.05 alpha level. Since the calculated F-ratio is less than the critical F-ratio, the null hypothesis is therefore not rejected; there is no significant difference in the counselling needs of adolescents with sickle-cell anaemia based on religion.

**Table 3:** Analysis of Variance (ANOVA) Showing the Respondents' Expression on Counselling Needs of Adolescents with Sickle-cell Anaemia Based on Religion

Source	df	SS	Mean Squares	Cal. F-ratio	Crit. F-ratio	p-value
Between Groups	2	23.538	11.769	0.84	3.00	.432
Within Groups	118	1645.024	13.941			

Total	120	1668.562				

## Discussion

The finding of the study revealed that the counselling needs of adolescents with sickle-cell anaemia include counsellors are expected to encourage adolescents with sickle-cell anaemia to have confidence in self. Adolescents with sickle-cell anaemia easily comprehend the counselling therapy of counsellor. Adolescents with sickle-cell anaemia are always willing to disclose self to the counsellor. Bediako, Lavender and Yasin's (8) findings stressed that sickle-cell disease is life-altering for most families; learning to accept, cope with and respond to this chronic illness requires that the counsellor and family work together. Cooperation occurs best in an environment where the family feels comfortable, safe and unjudged and the counsellor sets a tone for the relationship. That tone should encourage the family to view the counsellor as a resource, confidant and advocate. When working with children and families affected by sickle-cell disease, it is important to develop a comprehensive approach that encompasses psychosocial issues. Working to understand the issues faced by many of these families will help improve relationships and ensure a positive outcome.

The testing of hypothesis one revealed that there was a significant difference in the counselling needs of adolescents with sickle-cell anaemia based on gender. This was in line with Okon (9) who stressed that personal counselling is necessary at all stages of life. At the elementary school stage, opportunities should be given to pupils for their self-expression. Personal guidance at this stage deals with the problems related to feeling of insecurity, social acceptance, discipline, etc. At the secondary stage, the students have more intricate personal problems in relation to their gender.

The testing of hypothesis two revealed that there was no significant difference in the counselling needs of adolescents with sickle-cell anaemia based on religious affiliation. This negates Egbochuku (6) who opined that change is believed to arise from the provision of a supportive, empathic and validating therapeutic environment. In the traditional psychoanalytic approaches or the conflict model of psychopathology, behavioural disorder results from defences against conflicts. Thus, with respect to psychotherapeutic management of SCD crises, behaviour geneticists offer a series of coherent therapeutic approaches using traditional strategies such as confrontation, clarification, interpretation, and working through conflicts and painful crises, especially in transference situations.

In conclusion, the counselling needs of adolescents with sickle-cell anaemia include counsellors are expected to encourage adolescents with sickle-cell anaemia to have confidence in self. There was a significant difference in the counselling needs of adolescents with sickle-cell anaemia based on gender while there was no significant difference in the counselling needs of adolescents with sickle-cell anaemia based on religion.

Based on the finding of the study, the following recommendations were made:

1. The Government should offer standard healthcare for all adolescents with sickle-cell disease.
2. Counselling service should be provided as treatment modality for improving psychosocial functioning in adolescents with sickle-cell disease.
